# The Problem of the Mentally Defective

**Published:** 1913-02-22

**Authors:** 


### THE PROBLEM OF THE MENTALLY DEFECTIVE.

### Discussion at Central Poor-Law Conference.

The forty-first annual Central Poor-Law Conference was held at the Guildhall on Tuesday, February 11, and Wednesday, February 12. This year the first day's proceedings were particularly interesting, as papers were read on various aspects of the Mental Deficiency Bill. The Lord Mayor (Sir David Burnett) presided at the outset, and welcomed the delegates on behalf of the City Corporation. Afterwards Mr. Morton Latham, Chairman of the Farnham Board of Guardians, took the chair, and delivered his presidential address.

Major Leonard Darwin, President of the Eugenics Education Society, read a paper on "Mental Deficiency," in which he said that many persons seemed to find it impossible to understand that in such matters as the laws of heredity they were dealing with probabilities, and also that questions of probability could be discussed with mathematical exactitude. The legal and social system seemed to be designed actually to encourage the appearance of the mentally deficient. The parents of the normal child had to feed him and to supply him with boots, while as regards the mentally-deficient boy he was frequently carried to school, generally fed there, and often given boots. If the parents were so defective as to make them live a life proving them to be unfit to have the care of children they would often be relieved of all parental responsibility and expense under the Poor-Law Act.

Moreover, when thus familiarised with the idea of State aid, the workhouse would seem an economical and comparatively attractive place for lying-in. And, since it was now generally admitted that the size of the family did partially depend on economic conditions, it followed that this lessening the strain on the parents of defectives must have some tendency to promote the fertility of this class.

In this case, as in so many others, it was easier to recognise the evil than to suggest the cure. For, even if it be freely admitted that their efforts on behalf of the feeble-minded were attended with evil consequences, yet it soon became apparent that the eugenist must never suggest their entire abandonment. In the first place, such a step would run counter to all our highest moral impulses. Again, if the parents of the mentally defective were themselves, as they believed, on the whole below the average as regards mental qualities, then they would as a class be especially little influenced by any economic pressure that might be brought to bear on them. Lastly, it would be seen that their main hopes must rest on the systematic segregation of feeble-minded children, a method which was bound to lessen the strain on their parents. In considering what other methods might be employed in order to purify the race, the eugenist sees that his main object must be to increase the output of children in the upper half of humanity, not actually, but relatively, to the output in the lower half; for in this way the general level would be raised. It had been suggested that this result would be obtained by the State making adequate provision for all mothers and all children who stood in any need of it; for productiveness would thus be encouraged in future amongst the higher types in the same way as it was now being encouraged amongst the lowest. This proposal certainly was worthy of consideration, and must be examined in the cold light of eugenic reasoning. In considering any such reform, they should estimate the percentage of families who now did actually receive the State aid which it was proposed in future should be offered to or pressed on all applicants. Let it be assumed, merely for the sake of illustration, that it was ascertained that 10 per cent. of all families could not have their fertility increased by the reform in question because it would not bring them any further State aid; and let it also be assumed, as the assumption most favourable to the proposed reform, that all these families were below the average in civic worth. Then there would remain 90 per cent. of the families which might be influenced by the reform; and of these families 40 per cent. would obviously be below the average of the whole in civic worth and 50 per cent. above it. Would the additional State aid result in more additional children being produced in this 40 per cent. below the average, in which case the race would be injured, or in the 50 per cent. above the average, in which case racial progress would be promoted? That was the question which has to be decided. If it be assumed that there was some segregation of ability in the better-off classes, and if, as was obvious, the promise of State aid afforded a greater temptation to increased fertility in the poor than in the better-off classes, it seemed almost certain that such a reform would produce most effect in the lower half of humanity, and would thus be harmful from the eugenic point of view. On the other hand, if there was no such segregation of ability amongst the better-off classes there seemed to be no need for any such bribe to increased fertility amongst them. In short, it seemed probable that further State aid, indiscriminate as regards hereditary qualities, would merely increase, and not decrease the racial harm now being done; and in any case the probable results of such an experiment were so doubtful as to force eugenists to look elsewhere in search of a remedy.

It appeared, therefore, that no indirect methods thus far suggested of ridding the race of the curse of mental deficiency were likely to be effective, beyond no doubt continuing to hold in view the possibility of applying deterrent methods, especially such as, without causing actual suffering, marked the fact that independence was a higher ideal than dependence—as, for example, disenfranchisement; for they ought always to keep warring against that vicious tendency which our modern social methods had of making increased efforts to obtain State aid for the family appear more profitable than increased industry or forethought. But if they could place but very little reliance on indirect methods, ought they not to endeavour, by direct means, to check the further fertility of parents who had already produced mentally-defective offspring? If either of the parents was feeble-minded—and an inquiry in all such cases ought to be instituted—the eugenist would have no doubt that the State should step in and absolutely prevent further procreation. Again, where the stock from which the parents of the feeble-minded child had sprung could be proved to be very defective, or where more than one defective child has appeared, in such cases not only should all methods of persuasion be employed, but no relief, charitable or otherwise, should be given to such parents outside the workhouse as long as capable of bearing children.

But it must be admitted that, in comparison with the young and the defective, problems connected with the adult and the normal or nearly normal would ever remain more difficult to deal with from every point of view. Moreover, their institutional experience proves conclusively that a very large percentage of mental defectives remained remarkably contented under permanent control, however long it lasted, whereas freedom in their case often meant nothing but misery and disgrace. Their main eugenic hope, therefore, must for the present rest as regards mental defect on dealing with the feeble-minded child from infancy onwards in such a way as to prevent procreation when grown up.

Miss Emily C. Fortey, of Leicester, read a paper on "The Eugenic Aspect of the Mental Deficiency Bill." She said that only very extraordinary circumstances justified the setting aside of the fundamental rights of mankind. One of those rights was that of marriage including parenthood. If it were certain that the offspring would be degenerate, there would be a strong case for prohibition, but evidence showed that, even in the case of two feeble-minded parents, the children might be of normal intelligence. If legislation which aimed at preventing the procreation of the feeble-minded was desirable, arguments might be adduced in favour of State control of the member of a family and of the compulsory segregation of anyone who had a remarkably small head. If parenthood was forbidden on the ground that feeble-mindedness was transmissible to offspring it was surely essential to make certain that those whose condition was due to bad environment did not come under the prohibition. If that discrimination was not made—and the difficulties in its way were almost insuperable—a gross injustice was done to people whose only disqualification for parenthood was that they were very dull. Many were advocating nowadays that a certificate of health should be a necessary preliminary to marriage. Were they to supplement that by the test of an examination in scholarship? A measure which prohibited parenthood was a crude and clumsy method of dealing with an intricate problem of heredity. The statement was often made that the feeble-minded were increasing out of proportion to the rest of the population, but that was in direct contradiction to the evidence in the Report of the Lunacy Commissioners. If the Bill had passed into law, marriage with certain feeble-minded persons would be illegal. If a man who was morally weak had wronged a girl who was mentally weak, he would be debarred from making the only reparation in his power. In the name of womanhood she protested against making it illegal for a tardy act of justice to be rendered to one who might be an innocent victim and yet transmit to her offspring a heritage of shame. The Mental Deficiency Bill was unwarranted by present-day knowledge of the bearing of the laws of heredity on the question of feeble-mindedness. It must press unjustly on those who would not transmit their mental weakness to their offspring. Could they hope by selective breeding to bring about the physical, mental, and moral improvement of the race?

Mrs. Nott Bower, Chairman of the Richmond Board of Guardians, said that she would strongly deprecate the giving of permission to marry among mentally-defective people. She did a lot of work among fallen people, and had come to the conclusion that at least 50 per cent. of them were feeble-minded.

Lord Richard Cavendish said that the present system of training and educating the feeble-minded children was doing more harm than good. Either the period of detention should be indefinitely prolonged, or some more efficient system should be provided by which they could be looked after when they left the institution.

Mr. J. G. B. Stone (Canterbury) said that if the eugenist carried out his ideals to their ultimate conclusion, a lot of people would be stamped out before he had finished. His proposal would "put a premium on pedigree and make procreation a profession."

The Rev. H. Stanley (Great Yarmouth) urged that it was a disgrace to the country to talk about segregating these people and preventing parenthood.

The Rev. P. S. G. Propert, Chairman of the Fulham Board of Guardians, dealt with "The Administrative Aspect of the Mental Deficiency Bill." He suggested that, in the place of the new machinery proposed in the Mental Deficiency Bill, all the duties in connection therewith, and all the duties under the present lunacy laws now undertaken by the County and County Borough Councils should be undertaken by Boards, to be formed by the combination of suitable Poor-Law areas, generally co-terminous with the county acting, as at present, in concert with local Boards of Guardians. Such a proposal would simply enlarge the scope of work and the machinery already in existence. The authority, being composed of Poor-Law Guardians, would form a link between the Boards of Guardians throughout each district, and thus aid in promoting a far-reaching reform in Poor-Law administration in the combination. The number of such authorities would not exceed fifty-five, and by this means the Asylums Committees of 137 authorities would be disestablished, bringing great relief to many small county boroughs. The Mental Deficiency Bill, if carried in its present form, must lead to administrative confusion without effectively dealing with the problem. There was yet time to avoid that catastrophe, and he suggested that a scheme should be prepared, dealing with the whole body of the mentally defective by a simple extension of the lunacy laws and through one authority.

On Mr. Propert's recommendation the following resolutions were adopted:—(*a*) "That, in the interests of efficiency and economy, it is desirable that lunatics and other mentally-defective persons, liable to be sent to and detained in institutions or placed under guardianship by public authorities, should as far as possible be dealt with by one and the same authority"; and (*b*) "That the Mental Deficiency Bill now before Parliament should provide that all duties in connection therewith, and also those duties under the Lunacy Acts, shall be undertaken by boards of management of districts, to be formed by the combination of suitable Poor-Law areas acting in concert with the local Boards of Guardians."

### The Training of Poor-Law Children.

The business of the Conference was resumed on Wednesday morning, February 12, when the treatment and training of Poor-Law children was under discussion.

Mr. James M. Rendel (Kensington), in a paper on "The Urban Aspects of the Question," said that clearly the first object to be aimed at was to sever the children as early and as completely as possible from the workhouse and its traditions, and to give them a fair start in the race of life on equal terms with others. The Poor-Law school was, of course, not the only method by which that cardinal principle might be carried into effect. There was, for instance, the boarding-out system, which was very attractive, in theory at all events, and was warmly advocated as a practical expedient by many whose opinion was entitled to great respect. But the difficulties of working it were very serious. Suitable homes were hard to find, and there was always grave risk, even in spite of the most competent inspection, that the confidence of the Guardians would be abused and that the children would be exploited for the sake of the profit to be squeezed out of their maintenance allowance or their labour. It seemed obvious, therefore, that it could not be safely adopted on the scale required to meet the needs of the large towns. The "scattered homes" system was a kind of compromise between the boarding-out system and the school system, combining, in what proportion must depend on many circumstances, the merits and the faults of both. The economy claimed for it was perhaps illusory. If the cost of teaching did not appear in the Guardians' books it had to come out of the rates all the same. He believed that for London and the great cities the simplest and most natural expedient was the Poor-Law school, where the entire responsibility not only for the maintenance of the children, but also for their education and training, was directly assumed by the Poor-Law authorities. It seemed sometimes to be assumed as a matter of course that the children at such a school were specially handicapped, not merely by fortune but by nature, that they suffered in a peculiar manner from hereditary disability of one kind or another, and that they had more than their fair allowance of original sin. That was an entire mistake, and was proved to be so by the fact that according to the universal consent a very large proportion of the boys and girls educated in those schools turned out in after-life extremely well. If the expenditure on Poor-Law schools was thought to be excessive, by all means let them cut down luxuries and superfluities to any level of Spartan simplicity consistent with the needs of health, but when it came to education it must not be forgotten that there was nothing about those children which could be made an excuse for offering them instruction in any sense inferior to that which they would receive in the public elementary schools, and that any false economy in that direction was not only unjust to them but was bound to hasten the day when those schools would be taken out of the hands of the Guardians and transferred to the County Councils. While he did not for a moment suggest that the Poor-Law schools could be fashioned after the great public schools, if they kept such a type before them they would effectually exclude the image of an institution. There was in some quarters a good deal of prejudice against Poor-Law schools, which was summed up in the assertion that they turned out institution-made children, but that was not a true picture of any good Poor-Law school.

Miss Annette Louise Henry (Newbury), speaking as a country Guardian, said that there were still some country Unions which held to the old idea of the workhouse ward and sending the children to the ordinary elementary school of the district. That seemed a pity, for there were more suitable homes for boarded-out children than ever before. Poor-Law schools, cottage homes, and workhouse wards were unnatural homes and makeshifts at the best. Her Board always regarded with favour the wish of a lad to go to sea, as among the five thousand or six thousand tramps they annually got in their casual ward they had not found a seaman. It was a curious fact that Poor-Law children were about a standard lower in schools than other children of the same age. ("No!") Of nearly three hundred children who had passed through their hands in eighteen years they had had only five who had turned out badly.

After discussion a resolution was unanimously passed urging on the Local Government Board that no time should be lost in giving sanction to the proposals from combinations of Guardians for dealing with the feeble-minded cases.

### The Problem of the Vagrant.

Sir William Chance contributed a paper on "Vagrancy," in which he said that after all the efforts made to reduce the number of vagrants the country seemed to be worse off in regard to them now than at any time since 1868, when the question first occupied the attention of the Poor-Law Conference. The rise or fall of vagrancy used to be attributed to particular methods of treatment, but the only conclusion he could come to was that the more stringent regulations for the treatment of vagrants in the casual wards had not had so much effect as it was usually stated. Might not the rise of vagrancy be attributed to some extent to the Workmen's Compensation Act? Before the passing of that Act employers had not to be so particular as they had been since as to the age and physical condition of their workmen. The Factory and Workshop Consolidated Act had had the same effect, while the Unemployed Workmen's Act, which hardly anybody had a good word for, had certainly not been the means of rescuing any persons from the life of vagrancy. The Old Age Pensions Act hardly touched their subject. Some people seemed to expect a good deal from the Labour Exchanges Act in the matter of reducing vagrancy. He fancied, however, that the opinion universally held was that, while the Act helped the workman who really wanted work, it was practically useless as a help to the casual worker, from whose ranks the vagrant was drawn. A great deal was expected from the Insurance Act, but it was, of course, impossible to judge of its effects on vagrancy as yet. It should prevent the *bona-fide* worker temporarily out of employment from giving up hope of getting some other work, and so taking to the road in his despair, but it was doubtful whether it would make any very appreciable difference in the number of their vagrants. Modern trade unionism had probably had much to do with the increase of vagrancy of late years.

It had been said truly that if people would only button their pockets tightly against the appeals of tramps for "charity" a diminution of the numbers would soon follow. If the way and food ticket system were generally adopted, and the public learnt that the bodily wants of all vagrants were provided for, they might rightly expect that encouragement to vagrancy to come to an end. The difficulty of dealing effectively with tramps who had learnt all the tricks of vagrant life was much increased by the short sentences inflicted by some magistrates. He was doubtful whether labour colonies would suit the conditions of our country, but a State penal colony would undoubtedly be advantageous. It would be used solely for the habitual vagrants convicted as such at Quarter Sessions, and would form a part of our general prison system. This country had struggled with the vagrant for centuries, and he had always come out of the contest victorious. He doubted whether they would ever conquer him, but it was possible that they might get the upper hand in the end if the remedies suggested by the report of the Departmental Committee were tried without the necessity of transferring him from the care of the Guardians to that of the police. They did not want it any longer said that "between the Poor Law and the police the vagrant has flourished. The police authorities treat the vagrant as a criminal and do not punish him, while the Poor-Law authorities treat him as a pauper but do not relieve him."

On the motion of Sir WILLIAM CHANCE a resolution was adopted approving of the recent order of the Local Government Board relating to the better administration of relief to casual paupers.

